# Complementary Feeding and Risk of Choking: A Survey Among Parents and Primary Care Pediatricians in Emilia-Romagna, Northern Italy

**DOI:** 10.3390/children12121587

**Published:** 2025-11-22

**Authors:** Lorenza Parini, Elisa Manieri, Elena Corinaldesi, Michele Torella, Paolo Bottau, Eleonora Laderchi, Dalila Periccioli, Alessandra Cavallo, Chiara Bontempo, Eleonora Battelli, Egidio Candela, Monica Fae, Cecilia Argentina, Marcello Lanari, Arianna Dondi

**Affiliations:** 1 Specialty School of Paediatrics, Alma Mater Studiorum, University of Bologna, 40126 Bologna, Italy; 2 Pediatric Unit, Ramazzini Hospital, AUSL Modena Carpi, 41124 Carpi, Italy; 3 Pediatra Libera Scelta Convenzionato ASL, 40065 Bologna, Italy; 4 Pediatric and Neonatology Unit, Imola Hospital, 40026 Imola, Italy; 5 Pediatric Unit, IRCCS Azienda Ospedaliero-Universitaria di Bologna, 40138 Bologna, Italy; 6 UO Pediatria Territoriale, Dipartimento Cure Primarie AUSL, 40123 Bologna, Italy; 7 Department of Medical and Surgical Sciences, Alma Mater Studiorum, University of Bologna, 40138 Bologna, Italy; 8 Paediatric Anaesthesia and Intensive Care Unit, Department of Women and Child Health, IRCCS Azienda Ospedaliero-Universitaria di Bologna, Polyclinic Saint Orsola, 40138 Bologna, Italy; 9 Pediatra Libera Scelta ASL Romagna, 47814 Bellaria-Igea Marina, Italy

**Keywords:** complementary feeding, baby-led weaning, baby-led introduction to SolidS, traditional spoon-feeding, autonomous responsive feeding

## Abstract

Background: Complementary feeding has traditionally relied on traditional spoon feeding (TSF), in which parents gradually introduce semi-solid foods under close supervision. More recently, Baby-Led Weaning (BLW) has become popular, promoting infant autonomy in handling solid foods. To address concerns regarding choking and nutritional adequacy, the Baby-Led Introduction to SolidS (BLISS) method was developed. Some families instead adopt autonomous responsive feeding, which combines structured guidance with respect for the infant’s self-regulation. Although concerns about foreign body aspiration (FBA) persist among caregivers and pediatricians, current evidence shows that, when conducted safely, BLW may not increase this risk compared with TSF. This study investigated the prevalence of complementary feeding practices and their perceived relationship to FBA, exploring perspectives of caregivers and primary care pediatricians in Emilia-Romagna, Northern Italy. Methods: Between March 2022 and May 2024, 149 parents and 126 pediatricians completed anonymous questionnaires. Results: Among parents, 67% initiated complementary feeding at ≥6 months; 43.6% reported autonomous responsive, 32.8% BLW, and 23.5% strict TSF. Pediatricians more frequently endorsed flexible approaches: 61.1% supported autonomous responsive feeding, 37.1% BLW, and 12.7% TSF. Notably, strict TSF was applied by 23.3% of parents, almost twice the proportion recommended by pediatricians. Suspected choking episodes were reported by 41.6% of parents but showed no significant association with feeding method or demographic factors. Conclusions: BLW and related flexible practices are increasingly adopted and, when implemented safely, may not increase FBA risk. Pediatricians appear to recommend BLW, or hybrid approaches, more often than parents apply them, suggesting possible gaps in communication and shared decision-making.

## 1. Introduction

The World Health Organization (WHO) defines complementary feeding as the process initiated when exclusive milk feeding no longer meets the nutritional requirements of the growing child, making the introduction of other foods essential [1]. This period represents a phase of heightened vulnerability, influencing food acceptance, the establishment of healthy habits, and the prevention of growth stunting and nutrient deficiencies. Inadequate complementary feeding may compromise motor, cognitive, and socio-emotional development, while also increasing the long-term risk of overweight, type 2 diabetes, and adult disability [2,3].

Complementary feeding typically begins around six months of age and continues until 24 months, influenced by neuromotor development, individual predispositions, maturation of renal and gastrointestinal functions, as well as social and socioeconomic factors [4]. According to WHO guidelines, the recommended age for introducing complementary foods is about six months, a time that coincides with functional maturation of the digestive and renal systems and reduced susceptibility to gastrointestinal infections [1]. Nonetheless, international guidelines continue to show some discrepancies regarding the optimal timing of introduction [5].

Until the late 1990s, the most widely endorsed approach was Traditional Spoon-Feeding (TSF), or Parent-Led Feeding, in which parents offer pureed foods with a spoon between 6 and 8 months, followed by gradual texture progression until the child begins eating family foods after one year [6]. In the following decades, however, concerns arose regarding whether TSF should remain the exclusive recommended approach. By around six months, most infants demonstrate sufficient neuromotor development to sit independently, self-feed, and coordinate chewing and swallowing [7]. Feeding methods may therefore affect a child’s food relationship and long-term growth and development [8,9,10,11,12].

From the early 2000s, responsive feeding models such as Baby-Led Weaning (BLW) gained prominence. In this approach, parents provide appropriate foods, but the infant regulates timing and intake, self-feeding small portions of the same foods consumed by the family [13]. A modified version, Baby-Led Introduction to SolidS (BLISS), was later proposed to address concerns about choking and inadequate nutrient intake, introducing strategies such as safe cuts and nutrient-dense food selection [14,15].

Evidence suggests that, compared with TSF, BLW may reduce food selectivity, promote satiety regulation, and support adequate growth and nutrient intake. It may also facilitate adaptation to textures and flavors, accelerate chewing and fine motor development, and ease the transition to the family diet [12,16]. Family meal participation further promotes social interaction, emotional well-being, and healthy eating habits [17,18].

Nevertheless, the potential risk of choking remains a central concern. Foreign body aspiration (FBA) occurs when an object obstructs the airway, impairing gas exchange. It is commonplace under two years of age and represents the leading cause of accidental death in infants under one year and the fourth leading cause among preschool children [19]. The incidence and prevalence of FBA in children are not systematically reported in national or international registries. However, data from single-center studies and multicenter reviews indicate that it is a relatively common pediatric emergency, with a prevalence of approximately 75% during the first years of life [20,21,22]. Food is the most common aspirated material in the 0–2 age group, with nuts (especially peanuts) being the leading cause of obstruction worldwide, followed by seeds and legumes [23,24,25,26]. Cultural, social, and educational differences further influence both feeding practices and exposure to high-risk foods [27].

According to BLW proponents, choking risk does not increase compared to TSF, as infants display a higher frequency of gagging—a protective reflex that prevents airway obstruction [13,28]. For example, Fangupo et al. [29] observed that BLISS-fed children gagged more often than controls at six months, with incidence declining by eight months, suggesting maturation of swallowing skills. Furthermore, infants who self-feed typically adopt more upright postures and remain more attentive during chewing, in contrast with TSF, where food is passively offered and often swallowed without adequate mastication [14,16].

Overall, when parents adhere to BLISS guidelines aimed at minimizing choking risks, aspiration rates are not higher than with TSF. A meta-analysis and two reviews confirm no association between BLW/BLISS and increased choking, indicating comparable safety between TSF and BLISS [30,31]. By contrast, a Turkish study [32] reported that in 80% of 417 bronchoscopy-confirmed aspiration cases under three years, the child was self-feeding, though nearly all aspirated foods were classified as high-risk until 36 months [33]. Similarly, Na’ara et al. [34] found that 44% of aspiration cases were caused by nuts or seeds, highlighting that the main determinant of risk lies in exposure to age-inappropriate foods and inadequate caregiver supervision rather than the feeding method itself.

The diversity of complementary feeding practices, combined with the lack of consensus on the optimal method, highlights the need for clear guidance that both accommodates the preferences of individual families and ensures infant safety. Despite international evidence, little is known about parents’ and pediatricians’ perceptions and practices regarding the choice of complementary feeding methods, the reasons underlying these choices, and how they are implemented. In this context, the present study aimed to assess the perspectives of parents and pediatricians, to identify prevalent complementary feeding practices in our region, and to explore perceived risks associated with different complementary feeding approaches.

## 2. Materials and Methods

### 2.1. Study Design and Setting

The present is an observational, cross-sectional, multicentric study conducted through the anonymous administration of two specific questionnaires addressed to: (a) the population of parents of children undergoing complementary feeding, and (b) the population of primary care pediatricians.

For the “parents” population, participants were recruited among parents of children admitted to the pediatric wards or attending the outpatient clinics or pediatric emergency departments of the participating hospitals, namely IRCCS AOU Policlinico Sant’Orsola in Bologna and Hospital of Carpi (Modena). All adult parents of children aged between 4 and 18 months who had already started complementary feeding were eligible. Participation required access to an electronic device with internet connectivity, comprehension of the Italian language, and provision of informed consent for study participation and data processing. Parents of children with chronic neurological disorders, dysphagia, chronic conditions requiring a specific diet, or intestinal diseases causing malabsorption were excluded. All eligible parents during the period of the study were invited.

For the “pediatricians” population, primary care pediatricians from the Local Health Units of Bologna, Imola, Rimini, Ravenna, and Forlì-Cesena were recruited, following the provision of informed consent.

Within the Italian National Health Service, the primary care pediatrician serves as the child’s first-line physician, ensuring preventive care, monitoring growth and development, and coordinating referrals to specialist services when required. The Emilia-Romagna region has an organized primary care pediatric system, which could be considered representative of the Italian National Health Service. The multicenter design allows for the inclusion of diverse local healthcare settings, enhancing the representativeness of the findings across the region and providing a robust overview of parental and pediatrician practices and perceptions regarding complementary feeding.

Each parent and each primary care pediatrician, after providing informed consent, supplied their email address to which the questionnaire was sent. The questionnaires were created using the REDCap platform, which enabled the generation of unique links for each participant, ensuring access to anonymous online surveys. The responses obtained were extracted and entered into two separate databases, one containing parents’ responses and the other containing pediatricians’ responses.

Data collection took place from March 2022 to May 2024.

In both questionnaires, respondents were asked to provide answers in two formats: “single choice” and “multiple-choice.” The questions addressed to parents investigated the following aspects: parental demographic data, including age, place of birth, and educational level; family composition, specifically the number of children aged between 4 and 18 months; child-related data, such as: age, sex, current weight and height, birth weight and length, gestational age, and age at the start of complementary feeding; complementary feeding data, including age at initiation, type (TSF, autonomous responsive feeding, BLW/BLISS), and reasons for the chosen method; type of foods offered to the child and dietary patterns followed by the family; feeding practices and complementary feeding methods adopted; parental opinions on BLW/BLISS; modes of food presentation (e.g., type of cut, method of preparation); information on possible episodes of suspected or confirmed food aspiration, defined as episodes characterized by coughing and/or respiratory distress occurring during complementary feeding (yes/no, frequency, type of food, and activities during which the episodes occurred).

The questions addressed to primary care paediatricians investigated: type of complementary feeding recommended to the parents of their patients and the underlying reasons; counselling methods (e.g., use of brochures, recommendations regarding foods to be avoided); type of guidance provided to parents for implementing complementary feeding (e.g., which foods, methods of preparation); opinions on non-traditional complementary feeding approaches.

For both populations, the types of complementary feeding were defined according to the following criteria:

TSF: characterized using commercial infant foods (e.g., freeze-dried preparations, purees, vegetable broth, cereals), administered in fixed doses, quantities, and schedules.

Autonomous responsive feeding: broadly following the traditional scheme but with flexibility regarding the timing of food introduction, doses, and schedules, relying more on parental choice than on strict guidelines.

BLW/BLISS: whereby the child is allowed to self-select both the type and quantity of foods, predominantly home prepared and offered during family meals.

### 2.2. Statistical Analysis

The responses obtained were extracted and reported in two separate databases: one for the parents’ responses and one for the pediatricians. The creation of a unique link sent to each email ensured that duplicate responses were not possible. Non-parametric variables were described as absolute and relative frequencies; parametric variables were expressed as means with standard deviations. All data analyses were performed using SPSS (IBM Corp. Released 2016. IBM SPSS Statistics for Windows, Version 24.0. Armonk, NY, USA: IBM Corp).

### 2.3. Ethical Statement

The study was approved by the local Ethical Committee on 17 March 2022 (protocol code: DIVPED 89/2022/Oss/AOUBo).

## 3. Results

### 3.1. Parents’ Population Questionnaire

For the parent population, 369 questionnaires were distributed, and 149 responses were received, yielding a response rate of 40%. Among the respondents, 96% were female, and 93.3% were born in Italy. Most parents (81.9%) had a university degree, while the remaining 18.1% had a high school diploma.

Among the children involved in the study, 92.6% were born at term; 1.4% (*n* = 2) were moderate preterm (32–33 + 6 weeks of gestation), and 6% (*n* = 9) were late preterm (34–36 + 6 weeks).

Regarding complementary feeding, approximately 60% of parents (*n* = 88) started when their child was six months old, while 7% (*n* = 10) when their child was at least seven months old and the remaining began before six months, mostly at five months (28.85%, *n* = 43) or four months (4%, *n* = 6).

Most parents (43.6%) chose an autonomous responsive feeding approach, following a traditional pattern while varying the method and timing of food introduction. Of the remainder, 32.8% opted for BLW/BLISS and 23.5% opted for a TSF.

We investigated the factors influencing the feeding choice (as reported in Table 1) and what motivates parents to choose one method of complementary feeding over another (as summarized in Supplementary Table S1). Among TSF adopters, more than half (57%) considered it the simplest and safest approach, 40% relied on specific infant foods due to lack of experience, and 20% viewed it as providing a healthier and appropriate diet.

Among parents choosing alternative methods, 52.6% perceived it as healthier, 56% as promoting a more varied diet, and 75.4% as providing diverse flavours and textures. Finally, almost a fifth of parents (18%) do not consider it appropriate to rely on the specific baby foods available on the market.

Two main aspects were found to be significant from the analyzed data: the family meal and the source of information referred to by the parent. Firstly, children sharing a meal with their family were more likely to self-feed (93.9% vs. 6.1%, *p*-value = 0.019), consistent with BLW principles. Secondly, 77% of TSF adopters had consulted their pediatrician, compared to 22.5% who had not, whereas 75.5% of BLW adopters did not consult their pediatrician beforehand (*p* < 0.001). Furthermore, completing a complementary feeding training course was associated with a preference for self-weaning or independent weaning over traditional weaning (*p*-value = 0.029). Comparing these data, a higher proportion of parents who did not consult their pediatrician had attended feeding courses, compared to those who only consulted their pediatrician (38.5% vs. 17.1%, *p*-value = 0.007).

A multinomial logistic regression model was performed (Table 2) to examine the associations between parental and contextual factors and the type of weaning adopted. The TSF was used as the reference category, with comparisons made for Autonomous responsive feeding and BLW/BLISS. Among the dependent variables, the parents’ educational background was significantly associated with both autonomous responsive feeding (*p =* 0.044) and BLW/BLISS (*p* = 0.029). This suggests that higher education levels increase the likelihood of belonging to these weaning categories compared with the TSF.

Sharing a meal with the family was significantly associated with BLW/BLISS (*p* = 0.011), indicating that the frequency of shared family meals influences the type of weaning adopted.

In contrast, consultation with the family pediatrician was inversely associated with BLW/BLISS (*p* = 0.001).

No statistically significant associations were found for gestational age, training course, or age at introduction of complementary feeding.

We compared the types of complementary foods offered, focusing on foods considered high risk for FBA, including grapes, nuts, fruit with seeds, raw apple, raw carrot, sausage, mozzarella cheese, seeds, and sweets. Foods such as grapes (*p*-value = 0.015), mozzarella cheese (*p*-value = 0.007), and orange segments (*p*-value = 0.008) were offered more frequently with the BLW/BLISS method than in other methods (Supplementary Table S2).

We also investigated parental reports of observed choking episodes, finding that 41.6% (*n* = 62) had witnessed at least one such event. In all the reported episodes, the child was under parental supervision, and no medical intervention was required. In four cases, the child was engaged in activities other than eating, such as talking, watching television, or playing.

The aspiration episodes were analysed in relation to gestational age at birth, child’s gender, age at introduction of complementary feeding, participation in family meals, attendance at complementary feeding course, and type of feeding method. None of these variables, including the type of complementary feeding, showed a significant association with reported aspiration episodes (Table 3 and Supplementary Table S3). Foods consumed during the episodes most frequently included bread (*n* = 9), crackers (*n* = 8), biscuits (*n* = 5), small pasta (*n* = 5), raw apple or pear (*n* = 6), pasta (*n* = 4), grated fruit (*n* = 3), meat (*n* = 2), and raw carrot (*n* = 2).

### 3.2. Paediatricians’ Population Questionnaire:

For the paediatricians’ population, 289 questionnaires were distributed, and 126 responses were received, yielding a response rate of 44%. The main results are summarized in Table 4.

Our data show that 61.1% pediatricians recommended autonomous responsive feeding, 37.1% suggested BLW, and 12.7% proposed TSF. Some pediatricians reported recommending more than one complementary feeding method from those indicated. Three pediatricians indicated ‘other’, describing practices that still align with autonomous responsive feeding, such as ‘moderate BLW,’ transitioning from TSF to BLW after the first month, or adapting feeding to the child’s nutritional needs and the family’s culinary traditions.

Most pediatricians reported a uniform approach to supporting parents: 76.4% investigate the family’s eating habits, 87.6% provide specific information to parents approaching complementary feeding, regardless of the recommended method. Two-thirds (66.6%) advise parents to avoid certain foods, especially those that pose an infection risk (e.g., honey, raw meat) and those high in sugar or salt, while 73% recommend safer cutting and food preparation techniques, such as strips, chunks, small squares, shredding, chopping, and slicing.

Among pediatricians who recommend TSF, the majority (73.7%) consider it the safest and most effective method at this developmental stage. However, 26.3% express concern about parents’ desire for autonomy in decision-making, and 15.6% believe that there is insufficient scientific literature on alternative methods. Conversely, almost half (49%) of pediatricians recommending non-traditional complementary methods consider the available scientific literature sufficient, 39.8% feel adequately trained, 59% view these methods as promoting parental autonomy, and 15.7% regard traditional approaches as outdated.

Regarding BLW specifically, 60% of pediatricians did not consider it associated with a higher risk of FBA. Over half reported that greater child autonomy during feeding positively influences both the child’s development (55.5%) and family mealtimes (67.4%). Conversely, about one-fifth (20.6%) believed that these methods could be a source of stress for parents (Supplementary Table S4).

### 3.3. Comparing Parents and Pediatricians

Table 5 reports a comparison of pediatricians’ suggestions and parental choices. While parental decisions regarding the timing of complementary feeding initiation appear to be consistent with pediatricians’ recommendations, statistical analysis revealed a significant discrepancy between parental practices and pediatricians’ guidance with respect to complementary feeding methods. Specifically, pediatricians were more likely to endorse flexible approaches than those implemented by parents (*p* value = 0.017). In contrast, TSF was recommended by only 12.7% of pediatricians, yet adopted by 23.3% of parents.

## 4. Discussion

### 4.1. Parents Choices and Pediatricians’ Suggestions

The data collected and analyzed in the present study allow us to highlight the preferences of pediatricians and parents regarding complementary feeding practices and to shed light on the motivations underlying these choices.

Our study confirms that complementary feeding methods involving a certain degree of child autonomy and greater flexibility, including BLW itself, are increasingly the predominant approaches adopted by parents and recommended by pediatricians [35]. However, there is no clear predominance of one method over another.

Parents who adopt BLW recognize its advantages, such as promoting a wider variety of foods and ensuring nutritional safety by avoiding commercially processed products. Although these products are subject to strict regulations to guarantee their safety (e.g., preservation techniques and the absence of chemical additives, artificial colourings, and preservatives), they still may contain added sugars or salt, which should be avoided or at least limited when feeding toddlers [36]. An analysis of the factors influencing the choice of complementary feeding revealed that a significant determinant is the sharing of meals with the family, in line with the principles of BLW. This is important as it provides the child with an opportunity to learn, to socialize, and to develop a positive relationship both with food and with the family context. This contributes to the child’s emotional well-being, and thus to their acceptance of a wide variety of foods, tastes, and textures. It is also important from a safety perspective, as infants must be supervised during meals; finally, it may help parents to better manage the prolonged self-feeding attempts [37].

Similarly, pediatricians who recommend more autonomous feeding methods believe these approaches provide significant benefits for both children and their families. They can improve eating habits across the household and enhance the overall family quality of life.

However, a notable discrepancy was observed between the feeding methods recommended by pediatricians and those practiced by parents. A significant proportion of parents (23.3%) prefer TSF, nearly double the proportion of pediatricians who recommend it (12.7%). In Emilia-Romagna, the primary care pediatricians are responsible for following the infant and their family from birth, including growth monitoring, breastfeeding practices, and the introduction of complementary feeding. Unlike other countries where specialized professionals may be involved, in this region, pediatricians typically provide all guidance on complementary feeding when the infant reaches the appropriate age and developmental milestones.

The discrepancy becomes particularly significant when considering parental decision-making. Many parents who adopt alternative feeding methods do so without consulting their pediatrician, instead relying on external sources, such as complementary feeding courses. Among parents who follow traditional methods, around half report insufficient training in complementary feeding and perceive BLW as risky and stressful. These concerns are shared by approximately 20% of pediatricians, who believe that BLW may have a negative psychological impact on parents.

Overall, these findings highlight challenges in the pediatrician-parent relationship and suggest that some parents seek guidance outside the healthcare system due to gaps in communication or perceived lack of support. Addressing these gaps could strengthen trust and collaboration, ensuring that professional recommendations are better understood and followed.

Other potential determinants, such as parents’ age, gestational age at birth, and timing of complementary feeding introduction, did not show a statistically significant association with the choice of feeding method. Nonetheless, some trends could be observed: infants introduced to solids at or after 6 months were more frequently found in the autonomous responsive and BLW/BLISS groups compared with TSF, while preterm infants were relatively more represented in the TSF group. Although not significant, these findings may reflect parental perceptions of greater safety and control when dealing with potentially more vulnerable infants, while delaying the introduction of solids seems to encourage more autonomous approaches.

Concerning the possible health determinants that may have guided the choice of one approach over another, we investigated the correlation between parental educational level and complementary feeding method. Parents holding a university degree were more inclined to adopt autonomous responsive feeding or BLW/BLISS compared with those with only a high school education, and this association is statistically significant after adjustment for potential confounders.

Sharing family meals was identified as a statistically significant predictor of adopting a BLW/BLISS feeding style, which may reflect the suitability of this approach for integrating the child into regular family mealtime routines.

Finally, having selected the feeding method following a strong recommendation from the pediatrician was negatively associated with the adoption of the BLW/BLISS approach, while no significant differences were observed between TSF and autonomous responsive feeding.

### 4.2. BLW and the Risk of FBA

One of the main concerns among parents and family pediatricians regarding alternative feeding methods is the risk of FBA and choking, as children are exposed to solid food from an early age. Pediatricians’ opinions on the risk are not unanimous.

However, most studies comparing children following the BLW method with those fed through the TSF have shown no significant increase in choking risk among BLW adopters. When complementary feeding is conducted with appropriate precautions, such as offering safe foods and maintaining close supervision during meals, the risk of FBA appears comparable across different feeding methods [30,31].

The findings of our study are consistent with the available literature [38]. Among parents, 41.6% reported at least one observed choking episode, with no significant differences across feeding methods (48.6% in the TSF group, 34.9% in the autonomous responsive feeding group, and 42.9% in the BLW/BLISS group; *p* value = 0.394). Although the number of reported episodes appears relatively high, it is important to emphasize that these were all parent-reported events, and none required medical intervention. It is often difficult to distinguish a mild choking incident from a true aspiration event, and no validated instrument currently exists to make this differential diagnosis. The occurrence of observed choking episodes was analyzed in relation to several variables; however, no significant associations were identified. Notably, in all reported cases, the child was under parental supervision, and no medical intervention was required, highlighting the importance of adequate vigilance during feeding. The risk, therefore, seems more related to unfamiliarity with BLW than to an actual increased danger. Parents who choose BLW are generally educated about recognizing and managing ‘gagging’, a normal protective reflex which should not be confused with choking. In this sense, BLW supports feeding autonomy without compromising safety, while also supporting oral motor skills and a greater awareness of chewing and swallowing abilities. Allowing infants to explore and chew food of different textures promotes neuromuscular coordination and facilitates the integration of swallowing and respiratory patterns, processes that are essential for the safe progression of complementary feeding. Supporting this, Brown’s study showed that children accustomed to more liquid or semi-liquid foods were at higher risk of choking when first introduced to solids, suggesting that gradual exposure to solid textures, as in BLW, may reduce this risk.

In addition, foods that may pose a risk, such as hard or small foods (e.g., raw carrots, whole grapes, or nuts), are less frequently recommended in BLW than in the conventional method, helping ensure safety across both approaches. Despite this, the data from our study show that some risky foods, such as grapes, orange segments, and mozzarella cheese, are offered more frequently in BLW than in the other methods. As noted above, this choice does not appear to increase the risk of observed choking episodes; however, the data indicate that parental awareness of potentially hazardous foods—still frequently introduced—should be further strengthened.

### 4.3. Study Limitations and Prospects

This study provides additional evidence supporting the BLW approach; however, some limitations should be acknowledged. First, the sample size was relatively small, and the questionnaire response rate was modest (approximately 40%). Although previous studies have indicated that ethnicity, cultural background, and social class may influence the timing of solid food introduction, such comparisons were not feasible in our study, as almost all the participating parents were of Italian origin. Moreover, episodes of suspected FBA were defined according to parental reports, which may have led to potential misclassification of some events.

However, several questions deserving further investigation arise, for instance, comparing the age at which the aspiration episode occurred with their frequency over time could help clarify whether BLW supports oral motor development. It would also be interesting to assess whether parental choices differ between firstborn and subsequent children, possibly reflecting greater confidence and awareness, and to explore whether training in paediatric airway obstruction management enhances parental safety and reassurance.

Finally, no adjusted analyses were performed in this study; therefore, potential confounding factors could not be examined. As this was a cross-sectional study, the analyses were limited to describing associations between the collected variables.

Lastly, the data highlight the importance of exploring the communication barriers that seem to exist in our region between parents and pediatricians to better understand the dynamics that may influence not only decisions on infant feeding, but child health in general. Addressing such barriers could help foster a trusting relationship in which parents feel supported, and pediatricians can provide personalized information reflecting both the family’s needs and current scientific evidence.

## 5. Conclusions and Final Considerations

In conclusion, although BLW may initially raise safety concerns, our study contributes to the current literature by demonstrating that complementary feeding methods promoting greater child autonomy, including BLW, are becoming increasingly popular with parents and are also being recommended by pediatricians. Importantly, these methods do not appear to be associated with an increased risk of observed choking episodes compared to the traditional method. What matters most, regardless of the feeding approach, is that proper guidelines for safe and adequate meal management are consistently followed.

There remain, however, on the one hand, parents who still do not feel safe during complementary feeding, and on the other hand, parents who follow dangerous behavior such as feeding food with a higher risk of aspiration.

Given these findings, the primary care pediatrician needs to assume an active role in supporting and guiding parents during this delicate period. This includes promoting appropriate health education, encouraging participation in certified training courses recognized by official organizations, and recognizing the different needs of both parents and children. The evidence emerging from this study highlights the importance of introducing age- and developmentally appropriate foods. Supplementing verbal guidance with written educational materials, such as brochures or infographics, may help reinforce key recommendations and improve adherence to safe feeding practices.

Overall, these findings suggest the need for structured programs aimed at improving parental knowledge and confidence, enhancing pediatrician-parent communication, and ultimately promoting safe, developmentally appropriate complementary feeding practices.

## Figures and Tables

**Table 1:** Factors that can influence the choice of complementary feeding among parents. TSF = tablespoon feeding, BLW = baby-led weaning, BLISS = baby-led introduction to solids, RR = risk ratio.

	TSF (%)	Autonomous Responsive Feeding *n* (%)	BLW/BLISS *n* (%)	RR (CI 95%)	*p*-Value
**Chosen type of complementary feeding**	43.6%	23.5%	32.8%		
**Parents’ age**				1.41 (0.8–2.5)	0.183
<35 years old	18 (53%)	36 (58%)	35 (71.4%)		
≥35 years old	16 (47%)	26 (42%)	14 (28.6%)		
**Level of education**				0.55 (0.3–1)	0.191
High school	10 (28.6%)	10 (15.6%)	7 (14.3%)		
University degree	25 (71.4%)	54 (84.4%)	42 (87.5%)		
**Share a meal with family**				2.03 (1.1–3.7)	**0.019**
Yes	24 (70.6%)	52 (81.3%)	46 (93.9%)		
No	10 (29.4%)	12 (18.8%)	3 (6.1%)		
**Gestational age**				1.62 (0.7–3.7)	0.443
37 weeks	31 (88.6%)	60 (92.3%)	47 (96%)		
<37 weeks	4 (11.4%)	5 (7.3%)	2 (2%)		
**Age at introduction of complementary feeding**				/	0.389
4 months	0 (0%)	4 (6.3%)	2 (4.1%)		
5 months	13 (39.4%)	17 (26.6%)	12 (24.5%)		
≥6 months	20 (60.6)	43 (67.2%)	35 (71.4%)		
**Consultation with the family pediatrician**				2.87 (1.4–5.9)	**<0.001**
independent choice	8 (22.8%)	23 (35.9%)	37 (75.5%)		
Decision shared with pediatrician	22 (62.9%)	36 (56.3%)	10 (20.4%)		
Strong suggestion by a pediatrician	5 (14.3%)	5 (7.8%)	2 (4.1%)		
**Training course on complementary feeding**				1.68 (0.7–3.7)	**0.029**
Yes	6 (18.2%)	13 (21.3%)	19 (41.3%)		
No	27 (81.8%)	48 (78.7%)	27 (58.7%)		

**Table 2:** Multivariable logistic regressions on determinants that can influence the choice of complementary feeding among parents.

	OR	95% CI	*p*-Value
**Autonomous responsive feeding vs. TSF**			
Parents’ age	2.63	1.48–9.45	0.944
Level of education	30.37	2.85–125.23	**0.044**
Share a meal with family	6.06	1.78–17.57	0.317
Gestational age	6.09	1.17–31.54	0.631
Age at introduction of complementary feeding	2.16	1.53–3.36	0.272
Consultation with the family pediatrician	0.57	0.28–1.29	0.144
Training course on complementary feeding	3.91	0.81–89.46	0.634
**BLW/BLISS vs. TSF**			
Parents’ age	2.30	1.53–4.74	0.434
Level of education	118.53	3.24–18,443.0	**0.029**
Share a meal with family	184.70	36.27–937.36	**0.011**
Gestational age	3.15	2.31–5.66	0.393
Age at introduction of complementary feeding	1.96	1.38–3.91	0.280
Consultation with the family pediatrician	0.16	0.05–0.40	**0.001**
Training course on complementary feeding	11.80	4.64–27.54	0.188

**Table 3:** Reported observed choking episode. TSF = tablespoon feeding, BLW = baby-led weaning, BLISS = baby-led introduction to solids, RR = risk ratio.

	Aspiration *n* (%)	Not Aspiration *n* (%)	RR (CI 95%)	*p*-Value
**TSF**	17 (48.6%)	18 (51.4%)		
**Autonomous responsive feeding**	22 (34.9%)	41 (65.1%)		
**BLW/BLISS**	21 (42.9%)	28 (57.1%)		
			0.72 (0.45–1.16)	0.394

**Table 4:** Factors that can influence the type of complementary feeding practices suggested by primary care pediatricians to children’s caregivers. TSF: traditional spoon feeding; BLW: Baby-Led Weaning; BLISS: Baby-Led Introduction to SolidS.

	N (%)
**Workplace**	
Bologna and Imola	68 (54%)
Rimini	33 (26%)
Ravenna, Forlì-Cesena, or Carpi	25 (20%)
**Recommended age at introduction of complementary feeding**	
4 months	4 (5%)
5 months	15 (18.75%)
6 months	61 (76.25%)
**Elements considered for starting solids**	
Child’s psychomotor development	83 (66%)
Child’s age or corrected age if premature	81 (64.4%)
Child’s interest in food	74 (58.9%)
Parents’ needs	5 (3.7%)
**Recommended type of complementary feeding**	
TSF	16 (12.7%)
Autonomous responsive feeding	78 (61.9%)
BLW/BLISS	47 (37.3%)

**Table 5:** Comparing parents’ and pediatricians’ choices. TSF = tablespoon feeding, BLW = baby-led weaning, BLISS = baby-led introduction to solids, RR = risk ratio.

	Suggested by a Pediatrician *n* (%)	Parental Choice *n* (%)	RR (CI 95%)	*p*-Value
**Age of complementary feeding begins**			0.95 (0.43–2.08)	0.243
4 months	4 (5%)	6 (4%)		
5 months	15 (18.75%)	43 (28.85%)		
6 months	61 (76.25%)	100 (67.15%)		
**Complementary feeding methods**			1.74 (1.13–2.68)	**0.017**
TSF	16 (12.70%)	35 (23.30%)		
Autonomous responsive feeding	78 (61.90%)	65 (42.60%)		
BLW/BLISS	47 (37.10%)	49 (32.65%)		

## Data Availability

The original contributions presented in this study are included in the article/Supplementary Material. Further inquiries can be directed to the corresponding author.

## Supplementary Materials

The following supporting information can be downloaded at: https://www.mdpi.com/article/10.3390/children12121587/s1, Table S1: Parents questionnaires; Table S2: Prevalence of Foods Administered During Different Types of Complementary Feeding; Table S3: Episodes of inhalation and associated variables; Table S4: Extract from the Pediatricians’ Questionnaire.
