# Supplementary material for: Complementary Feeding and Risk of Choking: A Survey Among Parents and Primary Care Pediatricians in Emilia-Romagna, Northern Italy
Source: Children (Basel). 2025 Nov 22;12(12):1587. doi: 10.3390/children12121587 (PMC12732239; doi:10.3390/children12121587)
Supplement: Supplementary file 1 [file children-12-01587-s001.zip › children-3921585-supplementary.pdf]

Supplementary Material

Supplementary Table S1. Parents questionnaires

**Traditional feeding**	**n**	**%**
It seemed the simplest and safest option	**20**	**57**
I had no experience, so I relied on specific pediatric food products	**14**	**40**
To offer a healthier and/or more physiologically appropriate diet	**8**	**22.8**
I was not aware of alternative approaches	**4**	**11.4**
It allows me to spend less time on food preparation	**3**	**8.6**
It seemed to be the best option	**11**	**31.4**
**Not traditional feeding**		
It allows for a more varied diet	**64**	**56**
It enables the provision of a healthier and/or more physiologically appropriate diet	**60**	**52.6**
It allows the introduction of a wide range of flavors and/or textures	**86**	**75.4**
It allows me to spend less time on meal preparation	**24**	**21**
It promotes earlier development of the child's autonomous eating	**48**	**42**
I do not want to follow the "rules of the market" (i.e., commercial interests)	**20**	**17.5**
Other	**14**	**12.3**

Supplementary Table S2. Prevalence of Foods Administered During Different Types of Complementary Feeding

	**Traditional n (%)**	**Autonomous n (%)**	**BLW n (%)**	**RR (CI 95%)**	***p*-value**
**Individual grapes**				0.55 (0.27-1.12)	0.015
Yes	27 (77.1%)	47 (73.4%)	22 (44.9%)		
No	8 (22.9%)	17 (26.6%)	27 (55.1%)		
**Nuts**				0.43 (0.18-1.03)	0.105
Yes	30 (85.7%)	44 (69.8%)	32 (65.3%)		
No	5 (14.3%)	19 (30.2%)	17 (34.7%)		
**Fruit with seeds**				0.68 (0.19-2.50)	0.816
Female	33 (94.3%)	58 (90.6%)	44 (91.7%)		
Male	2 (5.7%)	6 (9.4%)	4 (8.3%)		
**Raw apple**				0.92 (0.50-1.72)	0.930
Yes	11 (31.4%)	18 (28.1%)	15 (30.6%)		
No	24 (68.6%)	46 (71.9%)	34 (69.4%)		
**Raw carrot**				0.98	0.342

				(0.52-1.82)	
Yes	24 (68.6%)	47 (74.3%)	29 (60.4%)		
No	11 (31.4%)	17 (26.6%)	19 (39.6%)		
**Sausage**				0.85 (0.24-3.05)	0.540
Yes	32 (94.1%)	58 (90.6%)	46 (95.8%)		
No	2 (5.9%)	6 (9.4%)	2 (4.2%)		
**Mozzarella cheese**				0.62 (0.35-1.11)	0.007
Yes	19 (54.3%)	32 (50%)	12 (24.5%)		
No	16 (45.7%)	32 (50%)	37 (75.5%)		
**Seeds**				0.28 (0.07-1.09)	0.096
Yes	33 (94.3%)	50 (79.4%)	37 (77.1%)		
No	2 (5.7%)	13 (20.6%)	11 (22.9%)		
**Candies**				4.29 (3.20-5.76)	0.200
Yes	34 (97.1%)	64 (100%)	48 (100%)		
No	1 (2.9%)	0	0		
**Orange segments**				0.59 (0.33-1.04)	0.008
Yes	15 (42.9%)	23 (36.5%)	7 (14.3%)		
No	20 (57.1%)	40 (63.5%)	42 (85.7%)		

Supplementary Table S3. Episodes of aspiration and associated variables

	**Aspiration n (%)**	**Not Aspiration n (%)**	**RR (CI 95%)**	***p*-value**
**Gestational age**			1.14 (0.51-2.56)	0.990
≥ 37 weeks	4 (6.7%)	7 (8.1%)		
< 37 weeks	56 (93.3%)	79 (91.9%)		
**Age at the introduction of complementary feeding**			1.14 (0.36-3.65)	0.458
4 months	2 (3.3%)	4 (4.7%)		
5 months	21 (35%)	22 (25.6%)		
≥ 6 months	37 (61.7%)	60 (69.8%)		
**Sex**			1.25 (0.85-1.83)	0.331
Female	28 (45.2%)	47 (54.7%)		
Male	34 (54.8%)	39 (45.3%)		
**Share meal with family**			1.10 (0.67-1.79)	0.895
Yes	49 (81.7%)	73 (83.9%)		
No	11 (18.3%)	14 (16.1%)		
**Complementary feeding modality**			0.72 (0.45-1.16)	0.394

Traditional	17 (48.6%)	18 (51.4%)		
Autonomous	22 (34.9%)	41 (65.1%)		
BLW	21 (42.9%)	28 (57.1%)		
**Training course**			1.09 (0.70-1.72)	0.843
Yes	15 (25.4%)	23 (28.4%)		
No	44 (74.6%)	58 (71.6%)		

Supplementary Table S4. Extract from the Pediatricians' Questionnaire

**Do you believe that traditional weaning, compared to alternative methods up to baby-led weaning, may have positive effects on the parents who practice it?**
• I believe it can support parents psychologically and in everyday life: 43.6% (n = 55)<br>• The advantages may outweigh the disadvantages: 12% (n = 15)<br>• I don't believe it has any impact: 16.7% (n = 21)<br>• I believe it has a negative impact: 7.1% (n = 9)<br>• The disadvantages outweigh the advantages: 20.6% (n = 26)
**If you recommend changes to traditional weaning, do you believe these may have a positive effect on the child's development?**
• I believe it can significantly improve the child's development: 55.5% (n = 70)<br>• The advantages may outweigh the disadvantages: 17.5% (n = 22)<br>• I don't believe it has any impact: 13.5% (n = 17)<br>• I believe it mainly carries risks: 0.8% (n = 1)<br>• The disadvantages outweigh the advantages: 0.8% (n = 1)<br>• I don't know: 11.9% (n = 15)
**Do you believe that baby-led weaning, compared to traditional weaning, can improve shared mealtimes within the family?**
• I believe it can benefit the whole family, promoting shared meals and moments of daily life: 67.4% (n = 85)<br>• The advantages may outweigh the disadvantages: 3.2% (n = 4)<br>• I don't believe it has any impact: 13.4% (n = 17)<br>• The disadvantages outweigh the advantages: 12% (n = 15)<br>• I believe it may worsen shared meals, especially during certain stages: 4% (n = 5)
**Do you believe that modifications to traditional weaning, including baby-led weaning, may influence the risk of developing obesity and/or eating disorders during the child's development?**
• I believe it may help prevent the development of these conditions: 43.6% (n = 55)<br>• The advantages may outweigh the disadvantages: 9.5% (n = 12)<br>• I don't believe it has any impact: 28.6% (n = 36)<br>• I believe it may increase the risk of developing such conditions: 12% (n = 15)<br>• The disadvantages outweigh the advantages: 6.3% (n = 8)
